# The prognostic value of the myeloid-mediated immunosuppression marker Arginase-1 in classic Hodgkin lymphoma

**DOI:** 10.18632/oncotarget.12024

**Published:** 2016-09-14

**Authors:** Alessandra Romano, Nunziatina Laura Parrinello, Calogero Vetro, Daniele Tibullo, Cesarina Giallongo, Piera La Cava, Annalisa Chiarenza, Giovanna Motta, Anastasia L. Caruso, Loredana Villari, Claudio Tripodo, Sebastiano Cosentino, Massimo Ippolito, Ugo Consoli, Andrea Gallamini, Stefano Pileri, Francesco Di Raimondo

**Affiliations:** ^1^ Division of Hematology, AOU “Policlinico - Vittorio Emanuele”, University of Catania, Catania, Italy; ^2^ Division of Pathology, AOU “Policlinico - Vittorio Emanuele”, Catania, Italy; ^3^ Tumor Immunology Unit, Department of Health Science, University of Palermo, Palermo, Italy; ^4^ Nuclear Medicine Center, Azienda Ospedaliera Cannizzaro, Catania, Italy; ^5^ Division of Hematology, ARNAS Garibaldi, Catania, Italy; ^6^ Research, Innovation and Statistics Department. A. Lacassagne Cancer Centre, Nice, France; ^7^ Unità di Diagnosi Emolinfopatologica, IEO, Milano, Italy

**Keywords:** Arginase-1, Hodgkin lymphoma, PET-2

## Abstract

**Purpose:**

Neutrophilia is hallmark of classic Hodgkin Lymphoma (cHL), but its precise characterization remains elusive. We aimed at investigating the immunosuppressive role of high-density neutrophils in HL.

**Experimental design:**

First, N-HL function was evaluated *in vitro*, showing increased arginase (Arg-1) expression and activity compared to healthy subjects. Second, we measured serum level of Arg-1 (s-Arg-1) by ELISA in two independent, training (*N* = 40) and validation (*N* = 78) sets.

**Results:**

s-Arg-1 was higher in patients with advanced stage (*p* = 0.045), B-symptoms (*p* = 0.0048) and a positive FDG-PET scan after two cycles of chemotherapy (PET-2, *p* = 0.012). Baseline levels of s-Arg-1 > 200 ng/mL resulted in 92% sensitivity and 56% specificity to predict a positive PET-2.

Patients showing s-Arg-1 levels > 200 ng/mL had a shorter progression free survival (PFS). In multivariate analysis, PET-2 and s-Arg-1 at diagnosis were the only statistically significant prognostic variables related to PFS (respectively *p* = 0.0004 and *p* = 0.012).

Moving from PET-2 status and s-Arg-1 level we constructed a prognostic score to predict long-term treatment outcome: low s-Arg-1 and negative PET-2 scan (score 0, *N* = 63), with a 3-Y PFS of 89.5%; either positive PET-2 or high s-Arg-1 (score 1, *N* = 46) with 3-Y PFS of 67.6%, and both positive markers (score 2, *N* = 9) with a 3-Y PFS of 37% (*p* = 0.0004).

**Conclusions:**

We conclude that N-HL are immunosuppressive through increased Arg-1 expression, a novel potential biomarker for HL prognosis.

## INTRODUCTION

Hodgkin Lymphoma (HL) is a neoplastic disorder characterized by a peculiar tumour architecture with few, scattered neoplastic cells surrounded by accessory non-neoplastic reactive cells [1, 2]. These accessory cells are responsible for the persistent ^18^F-Fuoro-2-Deoxy-Glucose (FDG) uptake in positron emission tomography (PET) scan performed early after first two cycles of chemotherapy (PET-2) [3–6]. Currently, a positive PET-2 is the main predictor of standard treatment outcome in HL and, accordingly, clinical trials incorporating PET-2 in a risk-adapted strategy have shown promising results [7, 8]. Other clinically relevant prognostic factors in advanced-stage HL include the international prognostic score (IPS) [9], recently validated in a series of advanced-stage patients treated with ABVD (doxorubicin, bleomycin, vinblastine, dacarbazine) regimen [10] and the amount of tumour-associated macrophages (TAM) in the diagnostic biopsy [11].

Several studies have documented the prognostic role of a subset of myeloid cells, defined as “myeloid-derived suppressor cells” (MDSC) in several solid and haematological cancers. MDSC phenotype in humans is still controversial [12–16]. Our previous work found that MDSC are increased in HL and have a prognostic significance [17].

MDSC are a heterogeneous set of myeloid cells at different stage of maturation that eventually evolve to the stage of neutrophils with a phenotype largely overlapping that of the granulocytic series [18, 19]. MDSC exert an enforced immune suppressive function against T-lymphocytes, arising from high expression of arginase (Arg-1) [20–23]. In mature neutrophils, Arg-1 is constitutively produced and stocked in cytoplasmic azurrophil granules [24–26] to be readily released during inflammation, to deplete the milieu of arginine [22].

Arg-1 metabolizes L-arginine to L-ornithine and urea in cytosol [22]. In T-cells, Arg-1 induces depletion of arginine from the microenvironment, an essential amino acid for the effective function of T-cell receptor (TCR) zeta chain assembly and downstream signalling [23, 27] as a consequence, L-arginine depletion profoundly suppresses T cell immune responses and this has emerged as a fundamental mechanism of inflammation-associated immunosuppression [19, 28–31].

Since both leucocytosis and lymphopenia have been incorporated in IPS because prognostically relevant [9], we explored the functional activity of neutrophils in HL (HL-N), their relation with disease burden and the prognostic value of their product, Arg-1.

## RESULTS

### Neutrophils are dysfunctional and immunesuppressive in HL

**Figure 1:**
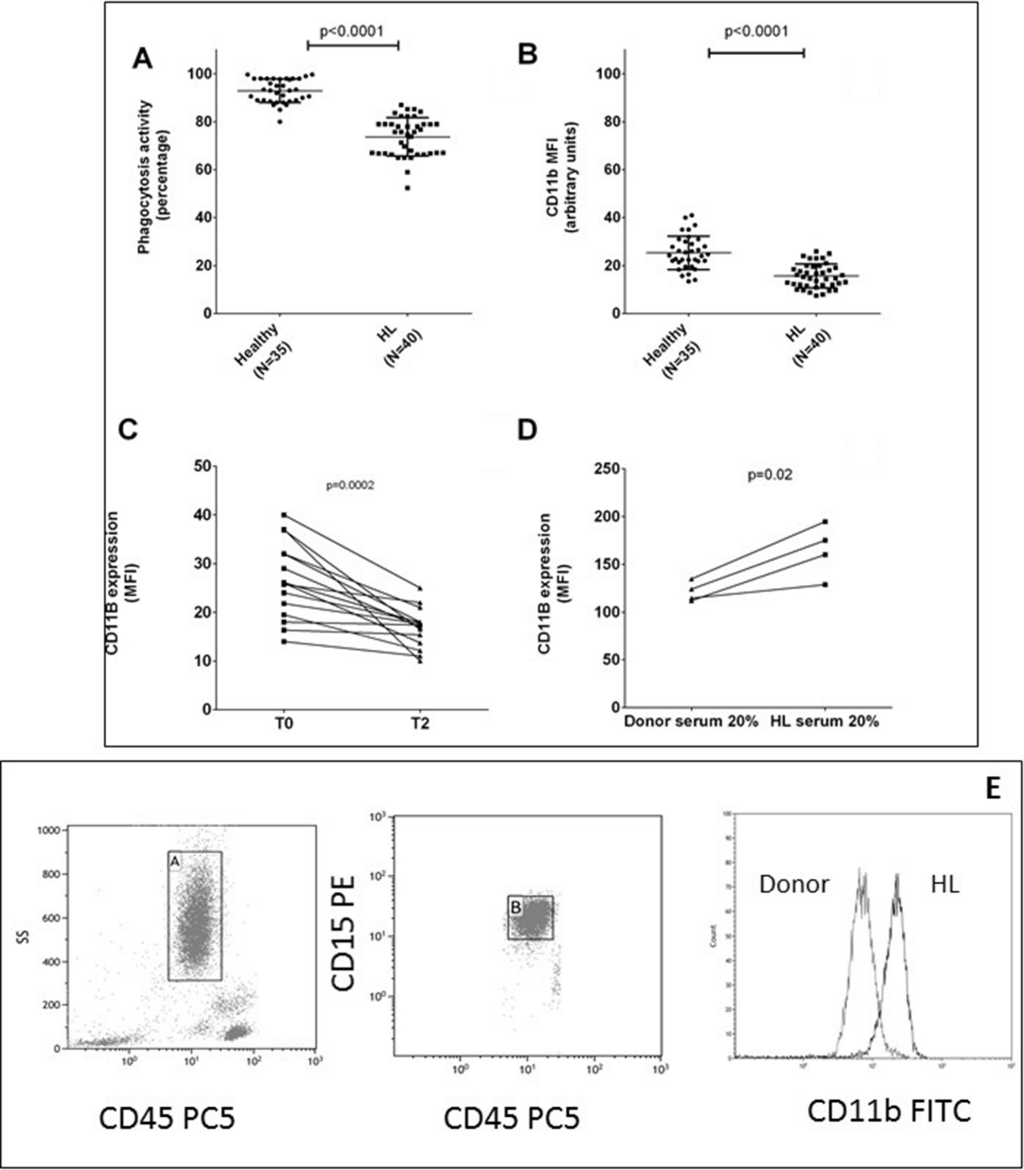
Neutrophils are dysfunctional in HL Mean values ± DS of phagocytosis test in neutrophils are shown from HL patients and healthy subjects in duplicate (panel **A**). Mean fluorescence intensity (MFI) of CD11b in neutrophils was evaluated at diagnosis (panel **B**) and after 2 courses of ABVD therapy (panel **C**). Neutrophils isolated from 4 healthy donors in two independent experiments were incubated for 24 hours with RPMI media plus 20% of serum obtained from healthy donor or HL patients in order to evaluate any change in CD11b MFI (panel **D**). Gating strategy for CD11b expression on neutrophils is shown in (panel **E)**.

Phagocytic activity was reduced in HL-N at diagnosis compared to healthy subjects (Figure 1A).

In order to understand if this finding was due to a persistence or lack of activation we investigated the neutrophil activation marker CD11b, because it promotes the phagocytosis of iC3b-coated particles and its expression increases rapidly following phagocyte activation. The MFI of the activation marker CD11b on HL-N was higher than CTRL-N (*N* = 40, 25.3 ± 1.3 versus 15.6 ± 0.8 au, *p* < 0.0001, Figure 1B) and reduced up to normal values after two courses of chemotherapy (as shown in 15 patients, 26.5 ± 1.1 versus 16.5 ± 1.0, *p* = 0.0002, Figure 1C).

In order to detect if a soluble factor, HL related, could explain these changes in N, we incubated for 24 hours 4 CTRL-N with serum obtained from HL or healthy donors and we observed that HL serum induced an increase of CD11b MFI (*p* = 0.02, Figure 1D).

**Figure 2:**
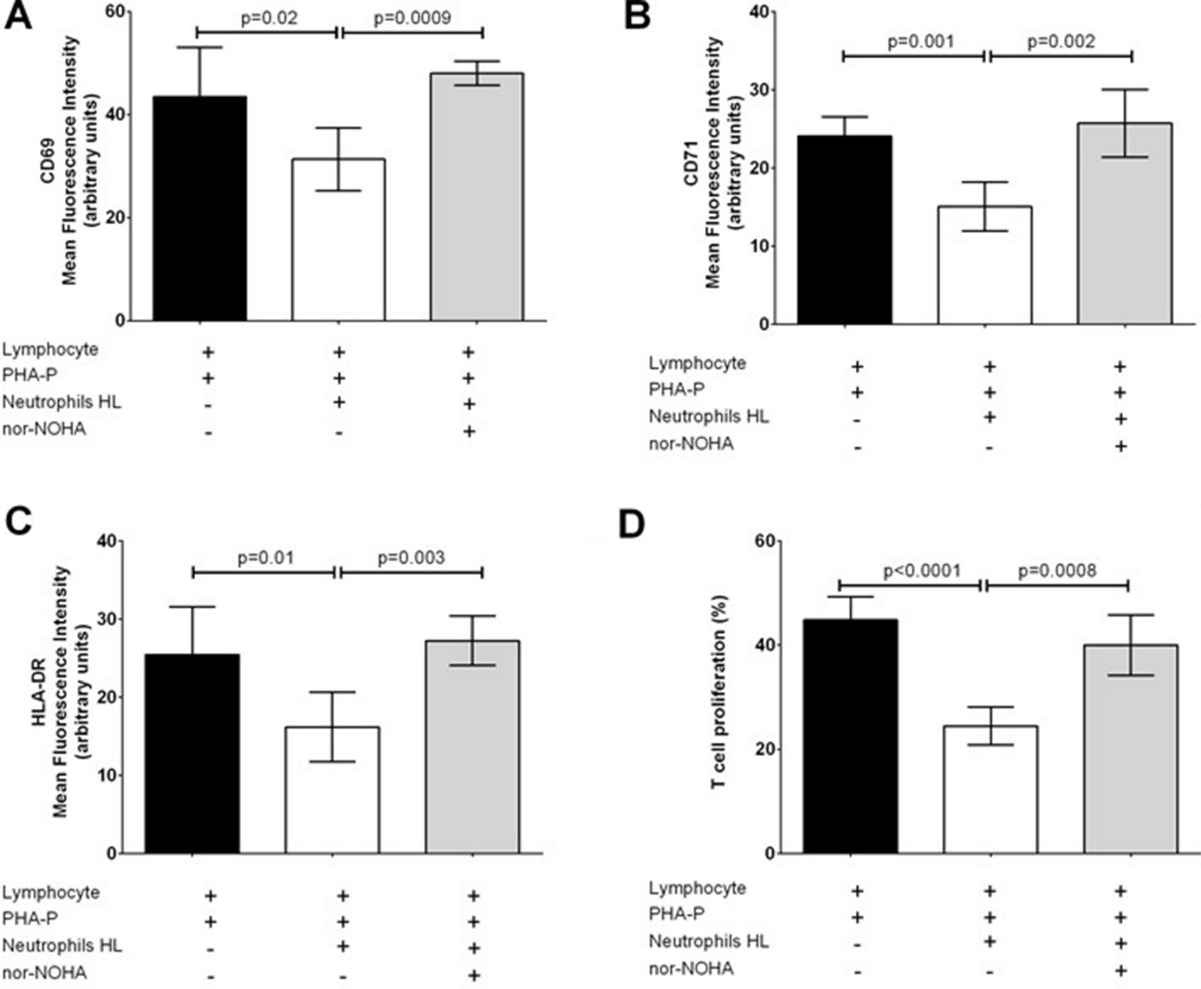
Immunosuppressive effect of HL neutrophils on T-cells can be reverted by nor-NOHA Results at 48 hours are reported separately for activation marker expression of CD69 (**A**), CD71 (**B**), HLA-DR (**C**) and proliferation (**D**) in h-Ly co-cultured with HL-N in absence (white bars) or in presence of 200 μM nor-NOHA (grey bars). Results represent MFI mean ± SD of duplicates from four donors and four patients, and are representative of three independent experiments.

Lymphocytes isolated from 5 healthy subjects (h-Ly) were co-cultured with neutrophils isolated from fresh peripheral blood of 8 HL patients (HL-N) and 5 healthy subjects (CTRL-N) in order to evaluate markers of activation after stimulation with PHA-P up to 72 hours in six independent experiments. In presence of HL-N, proliferation of T-cells was inhibited, in a dose-dependent manner (Supplementary Figure 2A). Similarly, despite PHA-P stimulation for 48 hours, CD69, CD71, HLA-DR and CD3ζ expression in h-Ly remained low after co-culture with HL-N, but not with CRTL-N, in a dose-dependent manner by increasing the ratio from 1:2 to 1:4 to 1:8 (Supplementary Figure 2B–2E). Treatment with 200 μM *nor-NOHA* (an Arg-1 inhibitor) for 48 hours reverted significantly this immunosuppressive effect, both at level of activation marker expression (Figure 2A–2C) and T-cell proliferation (Figure 2D).

### Arg-1 is increased in HL patients

Basic characteristics and treatments of both sets of patients are summarized in Table 1. Median age was 33 years (range 18–74) in the training set and 37 years (range 16–68) in the validation set. In both sets, most patients had advanced disease (Ann-Arbor stage ≥ IIB) and nodular sclerosis histotype. All except 5 had a history of infectious mononucleosis with positive antibody titre to the EBV viral capsid antigen.

**Table 1:** Patients’ characteristics

	Training set *N* = 40 (100%)	Validation set *N* = 78 (100%)	*p*-value
**Median age (range)**	33 (18–74)	37 (16–68)	
**Sex (M/F)**	24/16	48/30	0.98
**Bulky disease (Yes/No)**	11/29	11/67	0.08
**B-symptoms present (Yes/No)**	25/15	46/32	0.84
**Lymphocyte count/10^3uL median (range)**	1.33 ± 0.15	1.38 ± 0.31	0.51
**Neutrophil count/10^3uL median (range)**	8.58 ± 1.81	8.89 ± 1.63	0.35
**Ann Arbor stage**			
*Early disease*	9 (22.5)	28 (35.9)	0.14
*Advanced disease*	31 (77.5)	50 (64.1)	
**Hystotype**			
*Nodular sclerosis (%)*	30 (75)	60 (77.5)	
*Mixed cellularity (%)*	3 (7.5)	9 (12.5)	
*Lymphocyte-rich (%)*	7 (17.5)	6 (7.5)	
*Lymphocyte-depleted (%)*	0 (0)	3 (2.5)	
**IPS (only for advanced stage)**			
**< 2**	5	19	0.04
**2–7**	26	29	
**LDH median (range)**	386 ± 56	397 ± 47	0.26
**ESR median (range)**	45 ± 23	52 ± 31	0.21
**Treatment**			
ABVD 2 cycles ± i.f. radiotherapy	3 (7.5)	2 (2.5)	
ABVD 4 cycles ± i.f. radiotherapy	3 (7.5)	26 (33.5)	
ABVD 6 cycles	29 (72.5)	41 (52.5)	
ABVD 2 cycles + BEACOPP 8 cycles	5 (12.5)	9 (11.5)	

Clinical features of patients at baseline and their first-line treatment.

Abbreviations: LDH: lactico-dehydrogenase, ESR: Erythrocyte Sedimentation Rate, C-RP: C-Reactive Protein; i.f. involved field.

**Figure 3:**
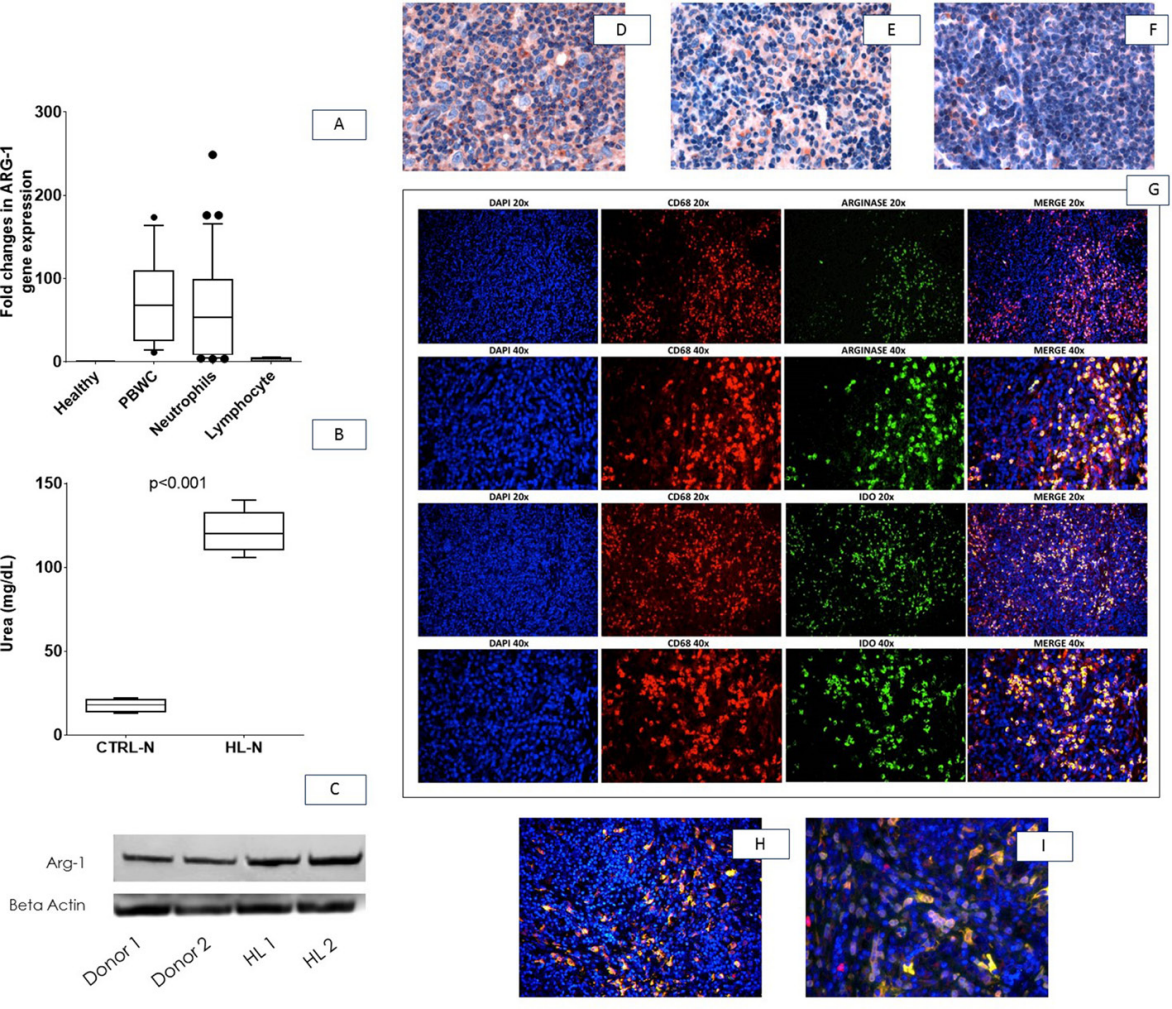
Arg-1 expression in neutrophils and lymph-node microenvironment Neutrophils isolated from control and HL patients were analysed for Arg-1 expression (**A**), activity by measuring urea (**B**) and protein expression by Western Blot (**C**). All assays were performed in triplicate. Representative microphotographs relative to Arg-1 *in situ* immunohistochemical detection in a case of mixed cellularity cHL (**D**), nodular sclerosis cHL (**E**), and in a lymph-node with reactive follicular hyperplasia (**F**). Double-marker immunofluorescence revealing that Arg-1^+^ cells within cHL environment were positive for CD68 (clone KP1, **G** upper panels) and IDO (Figure **3H–3I**) (20 and 40× magnification). Abbreviations: CTRL-N: pool of neutrophils from healthy subjects; HL-N: pool of neutrophils from Hodgkin's Lymphoma at diagnosis; Arg-1: Arginase 1

By RT-PCR, we measured ARG-1 in white blood cells (PBWC) and we found a marked increase of Arg- 1 expression of HL samples, almost entirely sustained by neutrophils (Figure 3A). Western blot of proteins obtained from HL-N confirmed this observation (Figure 3B). Neutrophils from HL patients showed also increased arginase activity in comparison to healthy subjects (Figure 3C).

To link the neutrophil dysfunction found in the peripheral blood of HL patients with the peculiar immunological milieu of the disease, we investigated the expression of Arg-1 *in situ*. Within the HL microenvironment, Arg-1-expressing cells with granulocytic and monocytic morphology were found intermingling with malignant cells (Figure 3D–3E). Arg-1+ cells were significantly enriched in HL tissue samples as compared with control reactive lymph node samples, in which only few scattered Arg-1-expressing elements were found within T-cell rich areas (Figure 3F). The myeloid lineage of Arg-1+ cells infiltrating HL were confirmed by double immunofluorescence analysis with CD68, clone KP1, which highlights myelo-monocytic elements (Figure 3G, upper panels). Moreover, the suppressor phenotype of Arg-1+ myeloid elements within HL microenvironment was also supported by double immunofluorescence for indoleamine 2,3-dioxygenase IDO (an enzyme acting as immune modulator through suppression of T-cell immunity, highly expressed in HL microenvironment and associated to poor outcome [32] which revealed that Arg-1+ cells also expressed IDO (Figure 3G, lower panels). Intensity of Arg-1 was independent from CD68 staining and clinical variables in the training set (data not shown).

**Figure 4:**
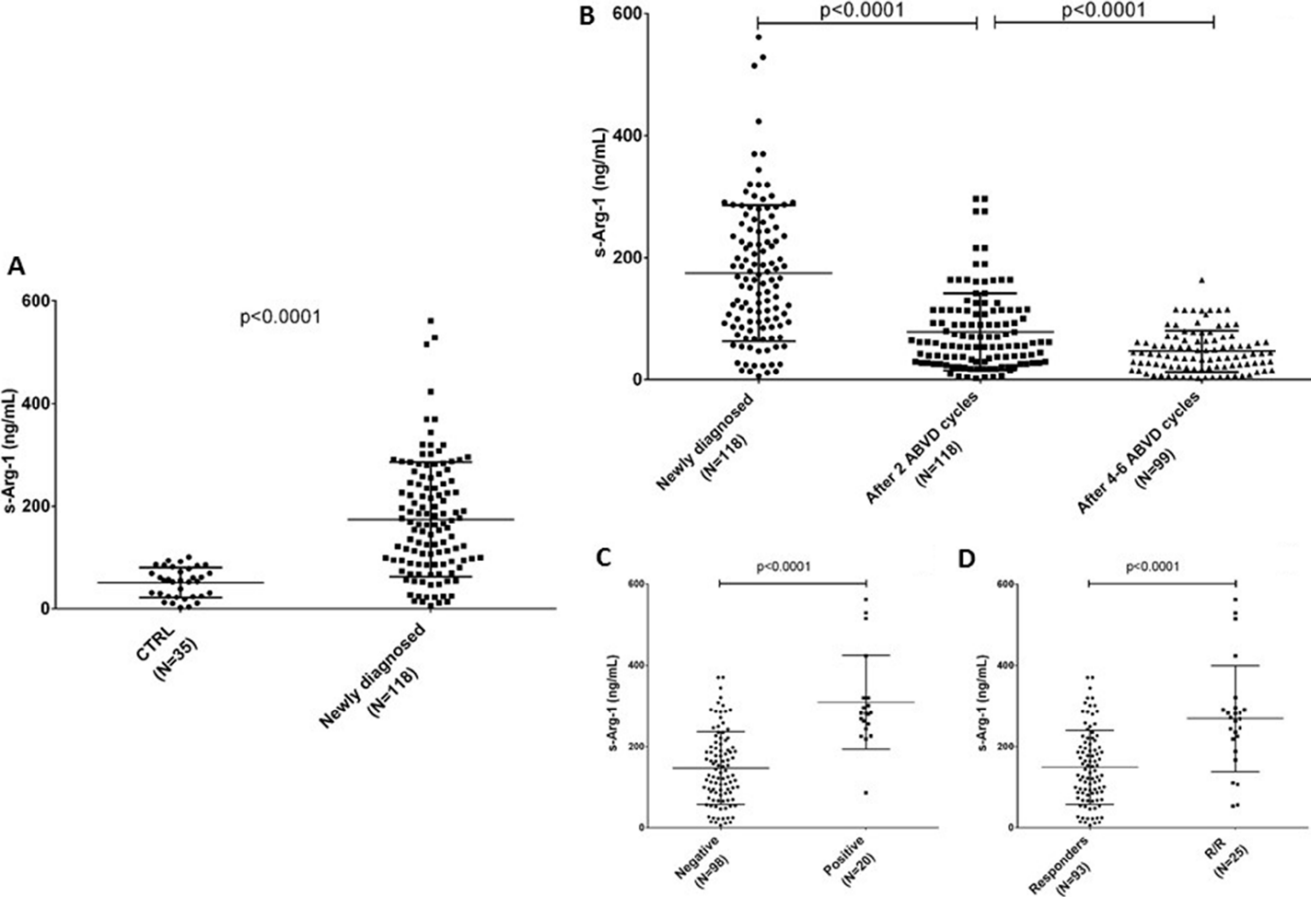
s-Arg-1 is increased in HL Concentration of arginase in serum (s-Arg-1) is reported for healthy volunteers and HL patients at diagnosis (panel **A**). Reduction in s-Arg-1 occurred by first two months of ABVD treatment, deeper at the end of planned treatment (**B**), except for PET-2 positive (panel **C**) or non-responder patients (panel **D**).

When we measured circulating Arg-1 in the serum of an enlarged cohort of patients (training+validation sets), we found it was increased in HL patients at diagnosis compared to healthy volunteers, mean ± standard deviation (SD) 174.6 ± 10.3 versus 51.5 ± 4.9 ng/mL (*N* = 118, *p* < 0.0001, Figure 4A). During and after standard first-line ABVD therapy Arg-1 was reduced: 78.6 ± 5.8 ng/mL after two cycles and 46.7 ± 3.4 ng/mL at the end of the planned cycles (Figure 4B, ANOVA *p* < 0.0001).

We then correlated the measurement of Arg- 1 in the serum at diagnosis with clinical findings. We found that patients with positive PET-2 showed higher Arg-1 at diagnosis compared to those who carried a negative PET2 (147.2 ± 9.1 versus 309.1 ± 25.8 ng/ mL, *p* < 0.0001, Figure 4C). Similarly, patients who achieved complete remission exhibited lower levels of Arg-1 compared to relapsed/refractory ones at diagnosis (149.2 ± 9.4 versus 269.2 ± 26.1 ng/mL, *p* < 0.0001, Figure 4D).

Arg-1 was increased in patients with advanced disease, B-symptoms, and elevated ESR in early-stage patients as shown in Supplementary Figure 3 for both training and validation sets.

### High Arg-1 amount is associated to shorter PFS

In the training set, 9/40 (22.5%) patients had s-Arg-1 at diagnosis higher than 200 ng/ml, and six of them had positive PET-2 and were addressed to an early salvage therapy accordingly to BEACOPP scheme, 3/6 achieving complete remission. A level of 200 ng/mL Arg-1 resulted in 87% (95% C.I. 71–96) sensitivity and 62% (95% C.I. 25–92) specificity in predicting achievement of complete response in the training set (area under curve, AUC, 0.81, *p* = 0.0078). Thus, a cut-off level of 200 ng/mL for s-Arg-1 was chosen to predict response status at 24 months (Table 2).

**Table 2:** Arg-1 at diagnosis can predict early (PET-2 status) and long term (relapse/refractory disease at 24 months follow-up) outcome of HL patients

	Training set (*n* = 40)			Validation set (*n* = 78)		
	Complete remission @ 24 months (N 32)	Relapse/Refractory disease (N 8)	R/R status @ 24 months	Complete remission @ 24 months (N 60)	Relapse/Refractory disease (N 18)	R/R status @ 24 months
Arg-1 < 200 ng/mL	**28**	**3**	*p* = 0.0078 Sensitivity 87 % (71–96) Specificity 62 % (25–92) PPV 90 % (74–98) NPV 56 % (21–86)	**44**	**3**	*p* < 0.0001 Sensitivity 73 % (60–84) Specificity 83 % (59–96) PPV 93 % (82–98) NPV 48 % (30–66)
Arg-1 ≥ 200 ng/mL	**4**	**5**		**16**	**15**	
	Complete remission @ 24 months (N 32)	Relapse/Refractory disease (N 8)	R/R status @ 24 months	Complete remission @ 24 months (N 60)	Relapse/Refractory disease (N 18)	R/R status @ 24 months
Negative scan	**30**	**4**	*p* = 0.009 Sensitivity 93 % (79–99) Specificity 50 % (16–84) PPV 88 % (73–96) NPV 67 % (23–95)	**55**	**8**	*p* < 0.0001 Sensitivity 92 % (82–97) Specificity 56 % (31–78) PPV 87 % (77–94) NPV 67 % (38–88)
Positive scan	**2**	**4**		**5**	**10**	
	Negative scan (N 34)	Positive scan (N 6)	PET-2 status	Negative scan (N 63)	Positive scan (N 15)	PET-2 status
Arg-1 < 200 ng/mL	**30**	**1**	*p* = 0.01 Sensitivity 88 % (73–96) Specificity 83 % (36–99) PPV 96 % (85–99) NPV 56 % (21–86)	**42**	**5**	*p* = 0.03 Sensitivity 67 % (54–78) Specificity 67 % (39–88) PPV 89 % (76–96) NPV 33 % (16–51)
Arg-1 ≥200 ng/mL	**4**	**5**		**21**	**10**	

We reported sensitivity, specificity, positive predictive value (PPV) and negative predictive value (NPV) in the training and validation set for Arg-1 as biomarker predictive of PFS at 24 months. 95% confidence interval are reported in round brackets.

In the validation set, baseline levels of s-Arg-1 > 200 ng/mL resulted in 73% (C.I. 95% 60–84) sensitivity and 83% (C.I. 95% 59–96) specificity in predicting response status at 24 months follow-up. Since the PET-2-oriented therapeutic approach, we evaluated also the ability of s-Arg-1 to predict PET-2 status, as reported in Table 2.

In the validation set, patients with high s-Arg-1 had shorter PFS at 24 months than patients with low Arg-1 (56.2% vs 93.4%, *p* < 0.0001, Supplementary Figure 4A), similarly to what described based on PET-2 status (28.7% vs 88.9 %, *p* < 0.0001, Supplementary Figure 4B).

**Figure 5:**
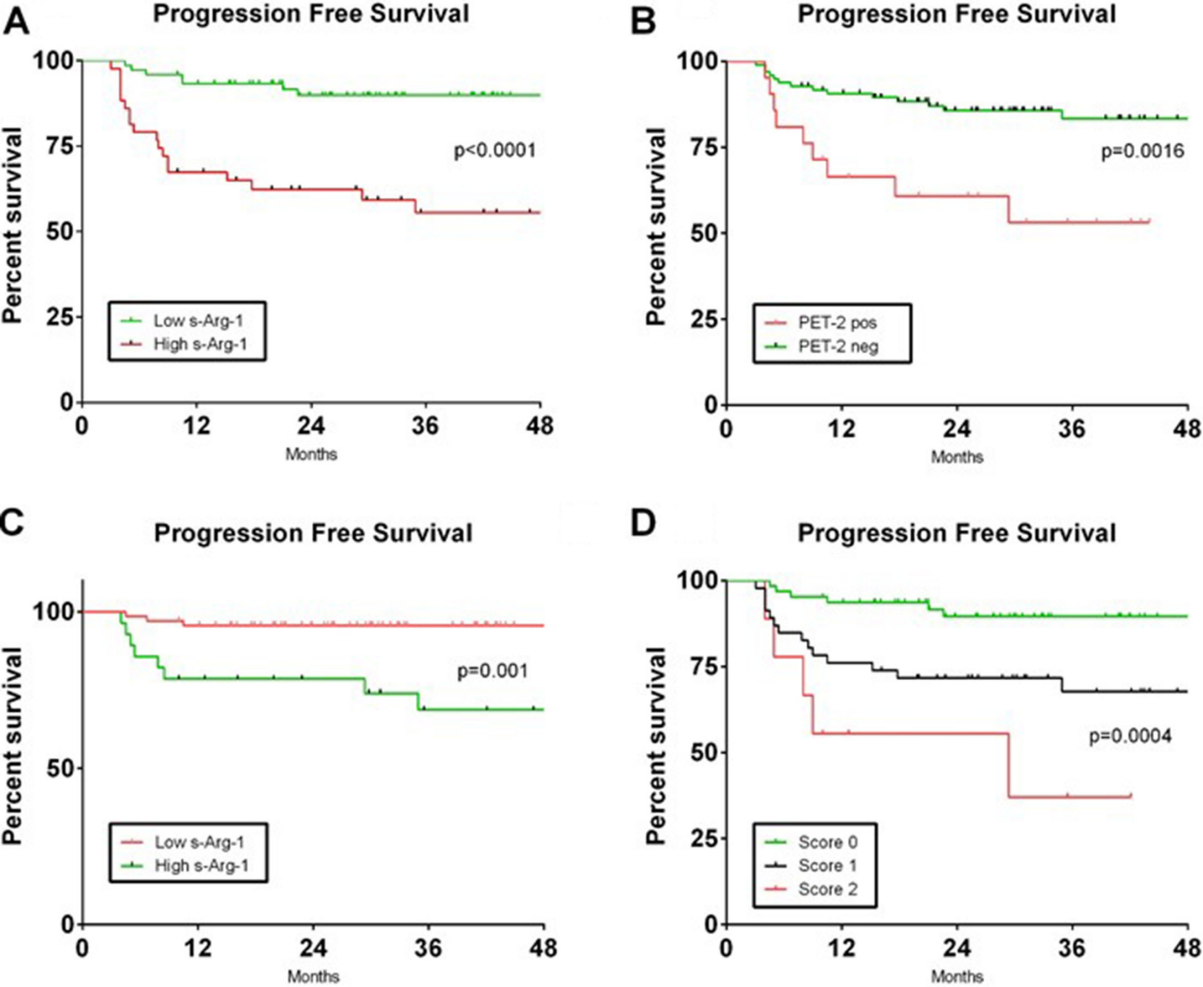
Progression free survival based on combination of PET-2 status and s-Arg-1 level Kaplan-Meier curves of progression free survival based on s-Arg-1 at diagnosis (panel **A**) and PET-2 scan (panel **B**) in the whole cohort of 118 patients are shown. Progression free survival in the cohort of patients carrying negative PET-2 based on s-Arg-1 is shown in (panel **C**). A score was developed based on PET-2 status after two cycles of chemotherapy and s-Arg-1 level at diagnosis (Panel **D**). Patients with low s-Arg-1 and negative PET-2 scan had score 0, patients with high s-Arg-1 or positive PET-2 scan had score 1 and patients with high s-Arg-1 and positive PET-2 scan had score 2.

Thus, in the whole cohort of 118 patients, PFS at 36 months was 89.8% for patients with low s-Arg-1 versus 55.5% in patients with high s-Arg-1 (Figure 5A). Based on PET-2 scan, PFS at 36 months was 83.3% in patients with negative scan and 53.2% in patients with positive scan, despite an early salvage treatment in patients with positive PET-2 scan (Figure 5B). In the group of PET-2 negative patients (*N* = 97), PFS at 36 months was 95.6% for patients with low s-Arg-1 (*n* = 69) versus 68.7% in patients with high s-Arg-1 (*N* = 18, *p* = 0.001, Figure 5C).

In multivariate analysis, PET-2 and s-Arg-1 at diagnosis were the only significant prognostic variables (respectively *p* = 0.0004 and *p* = 0.012, Table 3), despite the limited size of the sample and the adopted therapeutic strategy including early shift to BEACOPP treatment in case of PET-2 positivity.

**Table 3:** Cox proportional hazard regression analysis

Variable	HR	95%CI for HR	*p*-value
PET-2	4.6	2.1–10.8	*0.0004*
WBC > 15,000 cells/uL	1.6	0.28–13.3	0.58
s-Arg-1	3.3	1.3–8.4	*0.01*
B-symptoms	1.8	0.32–12.3	0.62

Abbreviations: HR hazard ratio, WBC: white blood cells.

With these two prognostic variables, we were able to define three distinct groups of patients based on PET-2 status after two cycles of chemotherapy and s-Arg-1 level at diagnosis. Figure 5D shows the PFS curves in patients with low s-Arg-1 and negative PET-2 scan (score 0, *N* = 63, 53%) or at least one unfavourable prognostic factors (score 1, *N* = 46, 40%) or high s-Arg-1 and positive PET-2 (score 2, *N* = 9, 7%).

Patients with score 0 had 89.5% PFS at 36 months versus 67.6% for patients with score 1 versus 37% for patients with score 2 (*p* = 0.0004).

## DISCUSSION

We previously showed that MDSC are increased in HL and have a prognostic role [17]. We therefore focused on neutrophils since neutrophilia is a common clinical finding in HL, and together with a low lymphocytes count is associated with a poor prognosis [9]. The imbalance between myeloid and lymphoid arm has been proposed as an additional biomarker in HL, including the ratio between the absolute counts of monocytes and lymphocytes [33]. However, the prognostic value of all the above markers was superseded by PET-2 [4].

In this work we investigated the function of neutrophils isolated from patients affected by HL sorted by physical methods. We found that N-HL are dysfunctional, since they exhibit a reduced phagocytic activity despite presence of activation signalling as shown by high levels of CD11b on the surface, as consequence of a sterile chronic inflammation leaded by soluble factors in HL including TGF-beta and IL-6 [34]. This uncoupled stimulation is likely due to one or more soluble factor(s) deserving specific investigation in further works.

We demonstrated that neutrophils of HL patients are able to reduce CD3ζ expression and other activation markers on the cell surface of T-lymphocytes stimulated with PHA-P (Figure 2), pointing toward an immunosuppressive activity of N-HL on normal lymphocytes. Downregulation of CD3ζ is a frequent mechanism of immunosuppression occurring in patients with solid cancers [19, 35–37]. The inflammatory environment may contribute to the accumulation of both mature neutrophils and MDSC. PMN from healthy donors activated with N-formil-L-methyonil-L-leucyl-L-phenylalanine co-purify with PBMC and suppress T-cells in a dose-dependent manner [38], similarly to mature neutrophils isolated from patients affected by sepsis [39] or mature neutrophils isolated from volunteers treated with LPS [34]. In absence of reliable markers to distinguish in human between normal mature neutrophils and G-MDSC, we hypothesized that aberrant function of HL-N could be due to a chronic stimulation (as suggested by CD11b expression) to sequentially downregulated T-cell function and inhibited effector responses during the transition to memory cells [40]. Indeed, we hypothesized that this immunosuppressive activity is mediated by Arg-1.

Arg-1 is contained in neutrophils, at the level of cytoplasmic azurophil granules as reported in most cases [24, 26, 41] but also in gelatinase grains [25]. Arg-1 modifies the metabolism of L-arginine, including the de-phosphorylation of cofilin, which is needed for the stability of the immunological synapse [42] and downregulates the CD3ζ chain translation in T cells, contributing to inhibit T cell proliferation [19, 22, 29–31].

In our study on neutrophil functional activity we showed that HL-N are characterised both by increased levels and activity of Arg-1 compared to healthy subjects (Figure 3). This is probably responsible for the increased Arg-1 serum levels that we have found in HL patients. In particular, s-Arg-1 levels proved higher in the serum of HL patients at diagnosis and promptly decreased upon effective treatment.

In both training and validation sets we found that Arg-1 is correlated to clinical features at baseline (stage, B symptoms, and elevated ESR). Most important, an Arg-1 serum level > 200 ng/mL at diagnosis represents a negative prognostic factor, able to predict response status at 24 months. In addition, we found that Arg-1 in some extend is able to predict PET-2 positivity, which remains, according to several recent reports the most important predictive factor on treatment outcome [3, 43–45]. This assumption has been also confirmed by our data, showing that a positive PET-2 may indeed identify patients candidate to a more aggressive treatment [45].

However, PET-2 information is available only during treatment and biological mechanisms leading to chemo-resistance may be activated as early as after 2 cycles of ABVD treatment. On the contrary, s-Arg-1 is a true prognostic factor, readily available at baseline before treatment onset, giving information that can be extremely useful for clinicians. Alternatively, it could be used as complementary information together with PET-2. Indeed, we found that Arg-1 and PET-2 can be combined in a prognostic score with three different probabilities of outcome.

In the last years, many studies have indicated the prognostic role in HL of serum cytokines, such as soluble CD30, IL-6, Il-2R or activation-related chemokine (TARC) [46]. However, different from the latter, Arg-1 is a specific marker of tumour functional activity as it portrays the T-lymphocyte suppression by HL. Future studies should explore the relationship of Arg-1 with other significant cytokines in HL to define a “cytokine fingerprint” in a single-patient basis with a strong potential prognostic impact.

In HL lymph nodes, high number of Arg-1^+^myeloid cells are significantly associated to shorter progression free survival in early stage patients and are correlated with a worse overall survival. In particular, a fraction of CD68KP1+ elements do also express Arg-1 in immunofluorescence [47]. Whether the prognostic value of MDSC Arg-1+ overlaps with that of TAM CD68KP1+ in HL [11] is still under investigation.

In conclusion, s-Arg-1 levels higher than 200 ng/mL are associated to a shorter PFS in HL. This observation needs to be prospectively confirmed in a larger series of patients.

## MATERIALS AND METHODS

### Patients

Peripheral blood samples were prospectively collected from 118 consecutive newly diagnosed patients treated at the Division of Haematology, AOU Policlinico-OVE, University of Catania between August 2012 and June 2014. First 40 cases were defined as training set (since these samples were used for functional evaluations), and further 78 patients considered as validation set. Thirty-five healthy subjects were evaluated as controls. The study was approved by the local IRB. All participants signed a written informed consent in accord to the Declaration of Helsinki.

The advanced-stage patients included in the present study were enrolled in the prospective multicenter phase II clinical trial HD 0607, as elsewhere reported [8]. Briefly, treatment started with 2 ABVD cycles [48, 49] and an interim PET (PET-2) was performed afterwards. On the basis of PET-2 results, patients with a positive scan shifted to a more aggressive treatment with BEACOPP regimen (bleomycin, etoposide, doxorubicin, cyclophosphamide, vincristine, procarbazine, and prednisone), four escalated + four baseline cycles; patients with a negative PET-2 continued with standard ABVD treatment [8]. Early stage patients received two or four courses of ABVD chemotherapy plus involved field radiotherapy as clinically indicated.

### FDG-PET scan assessment

Both baseline and interim PET were performed using standard scanning protocol. The scans were acquired at 60 ± 10′ (early) after the injection of the tracer and a semi-quantitative Deauville score was attributed to each image; scans were centrally reviewed by a panel of nuclear medicine experts in the HD 0607 trial, and by two local reviewers in early stage (I-IIA) disease [50, 51]. Only frankly positive and minimally positive scans were reviewed. A minimally positive scan was defined as any scan with any residual FDG uptake outside the physiological areas of the tracer concentration (mediastinal blood pool and liver) [43].

### Reagents

Ficoll-Paque was obtained from Pharmacia LKB Biotechnology. Heat inactivated/gamma-irradiated fetal bovine serum was obtained from Gibco Industries (Carlsbad, Ca). RPMI 1640 media with L-glutamine (1x), Dulbecco's phosphate-buffered saline (D-PBS) were purchased from Celbio, Italy and phytohemagglutinin (PHA-P) was obtained from Sigma-Aldrich (St. Louis, MO, USA).

The following anti-human antibodies were purchased from Beckman Coulter: HLA-DR- PC5 (Clone Immu-357), CD45 PC5 (clone J.33), CD15 PE (clone 80H5), CD3 ECD (clone UCHT1), HLA-DR- PC5 (Clone Immu-357), CD69 PE (clone TP1.55.3), CD71 FITC (clone DYJ1.2.2), CD11b FITC (clone Bear1) while CD3ζ/CD247 PE (clone 6B10.2) was purchased from eBioscience. Fluorescent-labeled isotype matched control antibodies were also purchased from Beckman Coulter, used as negative controls.

### Phagocytosis assay

Phagocytic activity of myeloid compartment was detected using the Phagotest kit (Opregen Pharma, Heidelberg, Germany).

Briefly, 100 μl heparinized whole peripheral blood is incubated with 20 μl opsonized FITC-labeled Escherichia coli for 10 min at 37°C in a water bath and in parallel a negative control sample remained on ice. The phagocytosis was stopped by placing the samples on ice. To eliminate the fluorescence of non-phagocytized bacteria, 100 μl of quenching solution were added. After a wash with 3 ml of washing solution (5 min, 250 × g, 4°C), the cells were incubated for 10 min on ice in 200 μl of DNA and analyzed by flow cytometry using a Coulter EPICS-XL-MCL cytometer.

Neutrophils were gated through the scatter parameters (forward, FCS vs side, SSC) and their green fluorescence histogram was analyzed. The phagocytic ability was expressed as percentage of fluorescent cells in the population studied and calculated by subtracting the percentage of the negative control sample (< 1%) from the positive sample.

### Isolation of neutrophils and lymphocytes

Whole blood (40 ml) was collected from healthy volunteers and HL patients in EDTA vacutainer tubes and diluted 1:1 with Dulbecco's phosphate buffered saline (PBS) (Celbio). Peripheral blood mononuclear cells (PBMC) were then isolated by density gradient centrifugation with Ficoll-Paque (Pharmacia LKB Biotechnology).

The resulting layer from the gradient was diluted and washed twice with PBS to obtain PBMC from the top and neutrophils from the bottom. T-lymphocytes were isolated using T-cells enrichment columns (R&D Systems) and their purity (> 90%) was assessed using flow cytometry.

To isolate neutrophils, the high-density layer was subjected to hypotonic lysis (155 mM NH4 Cl, 10 mM KHCO3, 0.1 mM EDTA, pH 7.4) for 15 minutes on ice. After washing, cell purity and viability were checked by flow cytometry (Supplementary Figure 1) and microscopy. PMNs showed a purity and viability of more than 90%.

### Evaluation of suppressive activity of neutrophils

To evaluate the suppressive activity of neutrophils, we assessed their ability to inhibit both proliferation and activation of T lymphocytes.

### T- cell proliferation

5 × 10^5^ of the T-lymphocytes isolated from 5 healthy donors were labelled with 1 μM of Carboxyfluorescein succinimidyl ester (CFSE) (BD Pharmingen) at 37°C for 20 min, then added to a 24-well tissue culture plate in the presence of phytohaemagglutinin (PHA-P, 5 mg/ml) (Sigma-Aldrich) and co-cultured with neutrophils (N) obtained from 8 HL or 5 healthy subjects (matched for sex and age) at ratio 1:2 and 1:8. The T-cell proliferation measured by CFSE dilution was evaluated by flow cytometry after 72 hours. The experiments were conducted in parallel in presence of 200 μM nor-NOHA.

### T -cell activation

In addition, T-lymphocytes from 5 healthy donors were harvested, washed twice in staining buffer (PBS containing 0.2% BSA and 0.1% sodium azide) and added to a 96-well polypropylene plate at concentration 5 × 10^5^ cells/well. After stimulation with 5 mg/ml PHA they were co-cultured with neutrophils (N) obtained from 8 HL or 5 healthy subjects at ratio 1:2 and 1:8.

Then, at 48 hours, we evaluated the expression on T-lymphocytes of some activation markers such as CD69, CD71, HLA-DR and CD3ζ. PHA-stimulated T-cells were used as control. The experiments were conducted in parallel in presence of 200 μM nor-NOHA.

Staining with respective isotype-matched control antibodies was also included for each condition to detect nonspecific background staining.

Density of expression of activation markers (Mean Fluorescent Intensity, MFI) were obtained and represented.

### Arginase assays

Neutrophils were plated at 5 × 10^5^ per well directly *ex vivo* in 96 well tissue culture plates, and washed with PBS and lysed with 0.1% Triton X-100 containing protease inhibitor (Roche, Nutley, NJ). To activate Arg-1, buffer containing Tris-HCl (25 mM) and MnCl2 (10 mM) was added and heated to 56°C for 10 minutes. L-arginine (0.5 M; Sigma) was added, and the samples were heated for 1 hour at 37°C. The hydrolysis of arginine was stopped with 800 μL of an acid solution mixture (H2SO4:H3PO4:H2O, 1:3:7). The amount of urea produced was determined using 9% α-isonitrosopropriophenone and compared with a standard curve with absorbance measured at 540 nm.

### Arg-1 detection by RT-PCR

Total RNA was extracted using TRIzol reagent and quantified using UV spectrophotometer. For real-time PCR analysis of mRNA expression, 1.0 μg of total RNA (in 20 μl reaction volume) was reverse transcribed using reverse transcriptase (Roche Diagnostic Corp., Indianapolis, IN, USA) and oligo-dT primers in a standard reaction. The quantitative real-time polymerase chain reaction was performed by use of a LightCycler (Roche), with primers specifically designed for Arg-1 (Forward:5′-CTCTAAGGGACAGCCTCGAGGA-3′, Reverse: 5′-TGGGTTCACTTCCATGATATCTA-3′; Applied Biosystem) according to the gene manufacturer's recommended protocol. Each reaction was run in triplicate. Samples were quantified accordingly (LightCycler analysis software, version 3.5) using the housekeeping gene GAPDH (Forward: 5′-CCAGCCGAGCCACATCGCTC-3′, Reverse: 5′-ATGAGCCCCAGCCTTCTC-3′; Roche) as standard.

### Western blot analysis

Briefly, for western blot analysis 30 μg of protein was loaded onto a 12% polyacrylamide gel Mini-PROTEAN^®^ TGX™ (BIO-RAD) followed by electrotransfer to nitrocellulose membrane Trans-Blot^®^ Turbo™ (BIO-RAD) using Trans-Blot^®^ SD Semi-Dry Transfer Cell (BIO-RAD). After blocking, membrane was three times washed in PBS for 5 minutes and incubated with primary antibodies against Arg-1 (anti-rabbit, Sigma-HPA003595). Next day, membranes were three times washed in PBS for 5 minutes and incubated with the secondary antibody FITC (anti-rabbit, Cat. No. sc-2012, Santa Cruz Biotechnology) for 1 h at room temperature. The blots were visualized using Odyssey Infrared Imaging Scanner (Licor) and protein expression levels were quantified by densitometric analysis of antibody responses. Data were normalized to protein expression levels of β-actin.

### Immunohistochemistry and immunofluorescence

Immunohistochemistry was performed on sections from four classical Hodgkin's lymphoma samples (2 nodular sclerosis and 2 mixed cellularity) and on sections from three lymph node samples of reactive follicular hyperplasia, using a polymer detection method as previously reported [52]. Briefly, tissue samples were fixed in 10% buffered formalin and paraffin embedded. Four-micrometers-thick sections were deparaffinized and rehydrated. The antigen unmasking technique was performed using Novocastra Epitope Retrieval Solutions pH6, pH 9 and pH 8 in PT Link Dako pre-treatment module at 98°C for 30 minutes.

Subsequently, the sections were brought to room temperature and washed in PBS. After neutralization of the endogenous peroxidase with 3% H_2_O_2_ and Fc blocking by a specific protein block (Novocastra UK) the samples were incubated over night with the primary polyclonal antibody anti-human Arginase 1, 1/200 pH 9 (GeneTex Catalog Number GTX109242), at 4°C. Staining was revealed by polymer detection kit (Novocastra) and AEC (3-amino-9-ethylcarbazole) substrate-chromogen. The slides were counterstained with Harris hematoxylin (Novocastra).

For double-marker immunofluorescence analyses, two sequential rounds of single immunofluorescence staining were performed. The following primary antibodies were used: polyclonal anti-human Arginase 1, 1/200 (GeneTex); monoclonal anti-Human CD68, 1/50 (clone KP1 Dako Denmark), monoclonal anti-Human IDO, 1/100 (clone 10.1, Novus biologicals).

After Fc blocking, primary antibodies binding was revealed by fluorochrome-conjugated secondary antibodies: Alexa Fluor 488 goat anti-Rabbit IgG (H+L, Invitrogen Molecular Probes, Carlsbad, CA), Alexa Fluor 568 conjugated goat anti-Mouse IgG (H+L, Invitrogen Molecular Probes, Carlsbad, CA).

Negative control stainings were performed by using rabbit immune serum instead of the primary antibody. The slides were counterstained with DAPI Nucleic Acid Stain (Invitrogen Molecular Probes). All the sections were analyzed under a Zeiss Axioscope A1 optical microscope (Zeiss) equipped with fluorescence module and microphotographs were collected using a Zeiss Axiocam 503 color (Zeiss).

### Arginase detection in peripheral blood

For sera collection, blood was centrifuged by 2 hours at 1600 × g for 10 minutes at room temperature and surnatant saved at −80°C for maximum 2 months. Arg-1 was detected in serum of healthy volunteer donors and HL patients by a commercial ELISA kit (Biovendor CS058) following the manufacturer's instructions.

### Statistical methods

Descriptive statistics were generated for analysis of results. Clinical and immunological parameters were compared by unpaired *t*-test. Baseline Arg-1 levels were correlated to Ann Arbor stage of disease, presence of B-symptoms and bulky disease, extranodal or bone marrow involvement and PET-2 status.

The cut-off of s-Arg-1 to identify patients with poor outcome was identified by the receiver operating characteristic (ROC) value with both sensitivity and specificity more than 70% in the training set to discriminate patients in complete remission versus refractory/relapsed within 24 months from the last chemo. This cut-off was applied in the validation set to compare the progression free survival (PFS) at 24 months between patients with high or not-high s-Arg-1 levels.

Progression free survival was calculated distinguishing high and not-high s-Arg-1 carrying patients according to Kaplan's Meier method.

Statistical analyses were elaborated through GraphPad Prism version 6.00 for Windows, GraphPad Software, San Diego California USA, www.graphpad.com.

## SUPPLEMENTARY MATERIAL FIGURES


