# Supplementary material for: The prognostic value of the myeloid-mediated immunosuppression marker Arginase-1 in classic Hodgkin lymphoma
Source: Oncotarget. 2016 Sep 14;7(41):67333–46. doi: 10.18632/oncotarget.12024 (PMC5341879; doi:10.18632/oncotarget.12024)
Supplement: Supplementary file 1 [file oncotarget-07-67333-s001.pdf]

The prognostic value of the myeloid-mediated immunosuppression marker Arginase-1 in classic Hodgkin lymphoma

Supplementary Materials

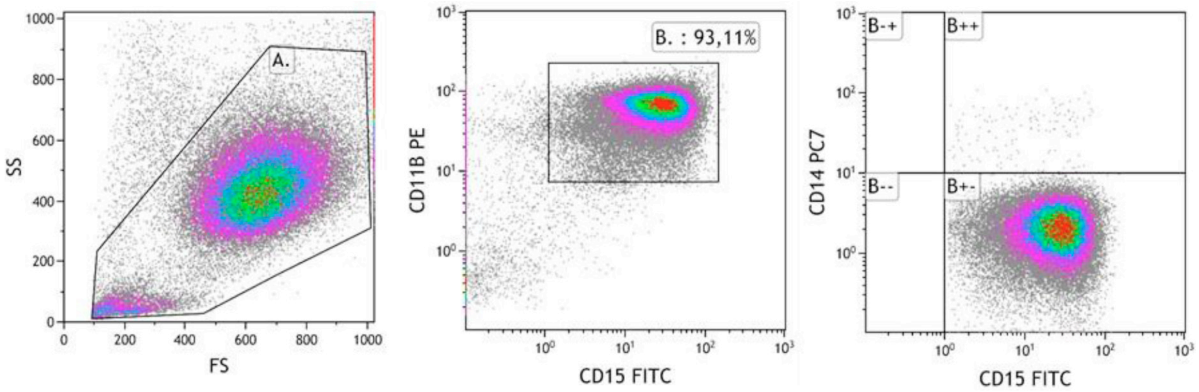

Supplementary Figure S1: Purity assessment after neutrophils isolation. Neutrophils were obtained after centrifugation of peripheral blood on Ficoll, followed by erythrocytes hypotonic lysis. Purity was checked by flow cytometry identifying $CD15^{+}CD11b^{+}CD14^{-}$ cells, ant it was always more than 90%. An example from a MM patient is shown.

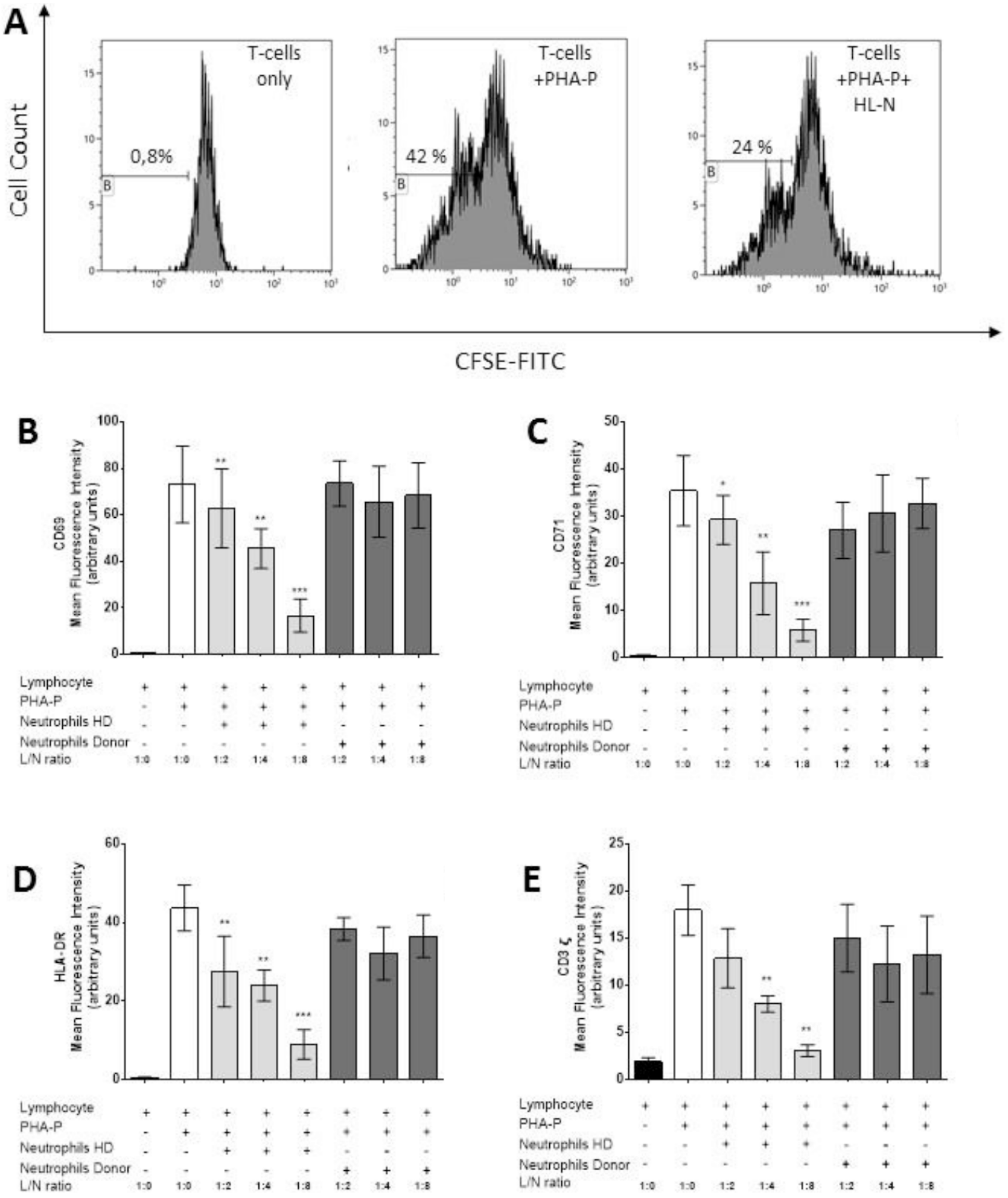

Supplementary Figure S2: Immunosuppressive effect of HL neutrophils on T-cells. Proliferation of T-cells from a representative experiment in presence of HL neutrophils at 1:4 ratio (**A**). Results at 48 hours are reported separately for activation marker expression of CD69 (**B**), HLA-DR (**C**), CD71 (**D**), CD3ζ/CD247 (**E**) in h-Ly co-cultured with HL-N (light grey bars) or CTRL-N (dark grey bars). Results represent MFI mean ± SD of duplicates from five donors and eight patients, and are representative of eight independent experiments. Abbreviations: h-Ly: lymphocyte from healthy volunteers; HL-N: neutrophils from HL patients; CTRL- N: neutrophils from healthy volunteers; L/N: lymphocyte/neutrophil ratio; PHA-P: phytohemagglutinin. *$p < 0.05$, **$p < 0.001$, ***$p < 0.0001$.

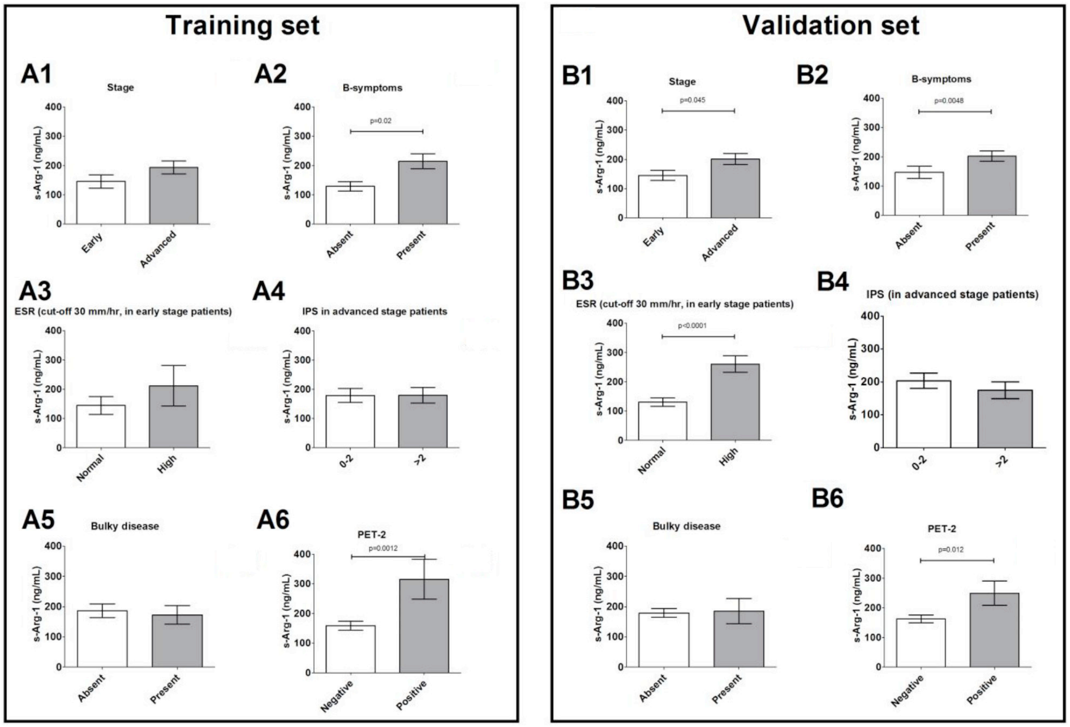

Supplementary Figure S3: Levels of s-Arg-1 and clinical features at diagnosis of HL patients.

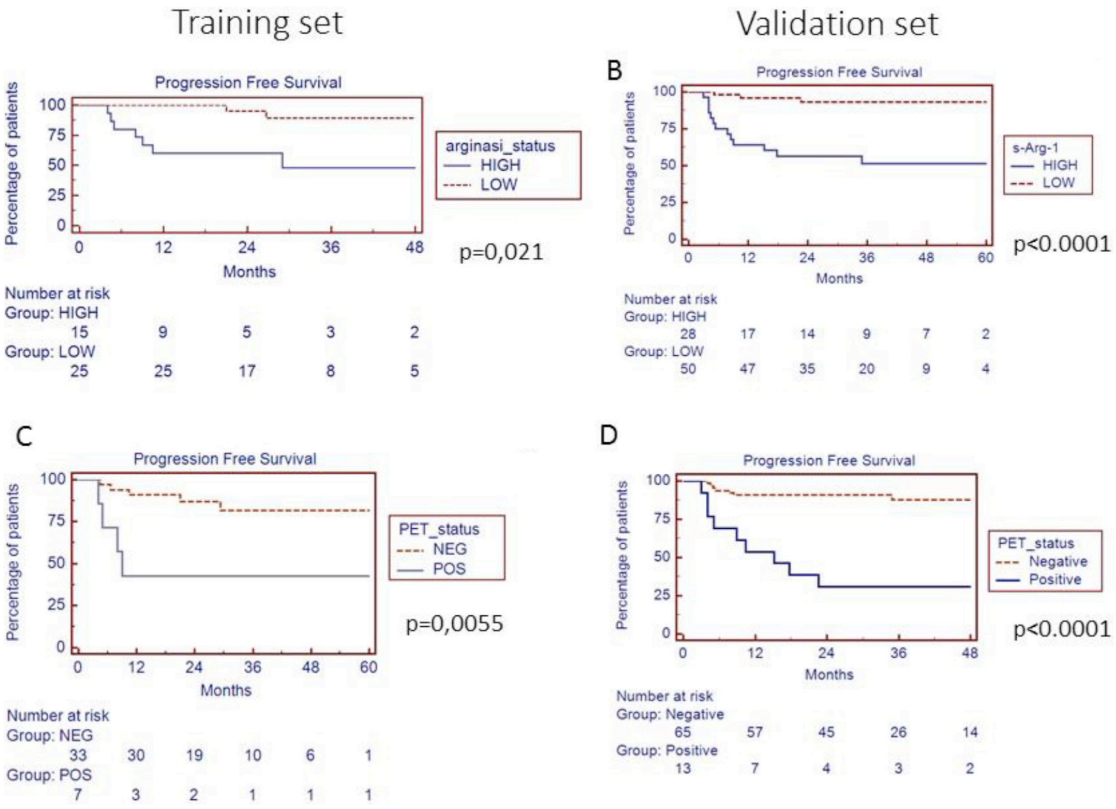

Supplementary Figure S4: Progression free survival based on s-Arg-1 at diagnosis and PET-2 9 scan in the training and validation set. Progression free-survival based on circulating s-Arg-1 at baseline in the training (panel **A**) and validation set (panel **B**); based on PET-2 scan in the training (panel **C**) and validation set (panel **D**).
